# The role of metabolic reprogramming in immune escape of triple-negative breast cancer

**DOI:** 10.3389/fimmu.2024.1424237

**Published:** 2024-08-13

**Authors:** Ruochen Bao, Hongtao Qu, Baifeng Li, Kai Cheng, Yandong Miao, Jiangtao Wang

**Affiliations:** ^1^ Thyroid and Breast Surgery, Yantai Affiliated Hospital of Binzhou Medical University, The 2^nd^Medical College of Binzhou Medical University, Yantai, China; ^2^ Emergency Department of Yantai Mountain Hospital, Yantai, China; ^3^ Cancer Center, Yantai Affiliated Hospital of Binzhou Medical University, The 2^nd^ Medical College of Binzhou Medical University, Yantai, China

**Keywords:** triple-negative breast cancer, immune escape, hypoxia, glucose metabolism, lipid metabolism, amino acid metabolism

## Abstract

Triple-negative breast cancer (TNBC) has become a thorny problem in the treatment of breast cancer because of its high invasiveness, metastasis and recurrence. Although immunotherapy has made important progress in TNBC, immune escape caused by many factors, especially metabolic reprogramming, is still the bottleneck of TNBC immunotherapy. Regrettably, the mechanisms responsible for immune escape remain poorly understood. Exploring the mechanism of TNBC immune escape at the metabolic level provides a target and direction for follow-up targeting or immunotherapy. In this review, we focus on the mechanism that TNBC affects immune cells and interstitial cells through hypoxia, glucose metabolism, lipid metabolism and amino acid metabolism, and changes tumor metabolism and tumor microenvironment. This will help to find new targets and strategies for TNBC immunotherapy.

## 1 Introduction

In 2020, breast cancer had the highest incidence worldwide and was the fifth leading cause of cancer-related deaths, accounting for one-quarter of female cancer cases and one-sixth of cancer deaths (1). Although the 5-year survival rate of patients with breast cancer has reached 80% (2), triple-negative breast cancer (TNBC) has emerged as a significant obstacle to improving the prognosis of patients with breast cancer due to its high invasiveness, propensity for metastasis, and recurrence (3). At present, surgery, radiotherapy and chemotherapy are still the main treatments for TNBC (4), but immunotherapy has shown promising results in the management of TNBC (5, 6), with a response rate of approximately 20% (7). Immune escape caused by many factors has become an important factor in the limited effect of immunotherapy for TNBC. Tumor heterogeneity and tumor metabolism play a pivotal role in this process.

Tumor heterogeneity is a key determinant of treatment efficacy and is associated with poor survival outcomes and prognosis (8, 9).There are various types of tumor heterogeneity. Foremost, heterogeneity may occur between individual tumor cells, known as intratumor heterogeneity. Intratumor heterogeneity can be further categorized into spatial and temporal heterogeneity, as it may be present in distinct regions of the primary tumor and the primary tumor may evolve over time. Secondly, the differences between different metastatic lesions in the same patient are called inter-metastatic heterogeneity. Thirdly, heterogeneity may also exist within a single metastatic cell. Finally, there is also heterogeneity between tumors of different patients, so personalized treatment is needed for each patient (10, 11).Tumor heterogeneity is ubiquitous in all cancers. In general, epigenetic modifications of cell characteristics such as tumor transcriptional changes, gene mutations, and changes in protein levels all show tumor heterogeneity. In addition to these intrinsic factors, other extrinsic factors such as pH, hypoxia, and crosstalk between tumor cells and other stromal cells in the TME can also affect tumor genotype and phenotype, further leading to tumor heterogeneity (12).

Metabolic reprogramming is a hallmark feature of tumors (13, 14), enabling tumor cells to meet the energy demands and biosynthetic requirements necessary for malignant proliferation, maintain redox homeostasis, proliferate rapidly in a nutrient-deficient tumor microenvironment (15), and facilitate the survival and metastasis of cancer cells (16). Alterations in metabolic reprogramming in tumor cells also impact immune cells and other cell types, contributing to tumor initiation and progression. In comparison to other subtypes of breast cancer, TNBC exhibits reduced mitochondrial oxidative phosphorylation, increased glycolytic activity, enhanced fatty acid synthesis, and altered amino acid metabolism (17–20).

Tumor heterogeneity plays an important role in tumor formation and development. Revealing tumor heterogeneity still faces many challenges. How to fully and comprehensively understand the changes in heterogeneity in patients is one of the future directions of tumor research. Due to the rich content of tumor heterogeneity and metabolic reprogramming, this review mainly analyzes the role of metabolic reprogramming in the immune escape of triple-negative breast cancer. This review starts from the mechanism of metabolic reprogramming in TNBC immune escape, and expounds that hypoxia, glucose metabolism, lipid metabolism and amino acid metabolism alter tumor metabolism and the tumor microenvironment by affecting immune cells, interstitial cells and other processes, resulting in immune escape. Indicating the importance of metabolic reprogramming in tumor progression, and then providing help for the discovery of new TNBC immunotherapy targets and strategies.

## 2 Hypoxia and immune escape in TNBC

**Figure 1:**
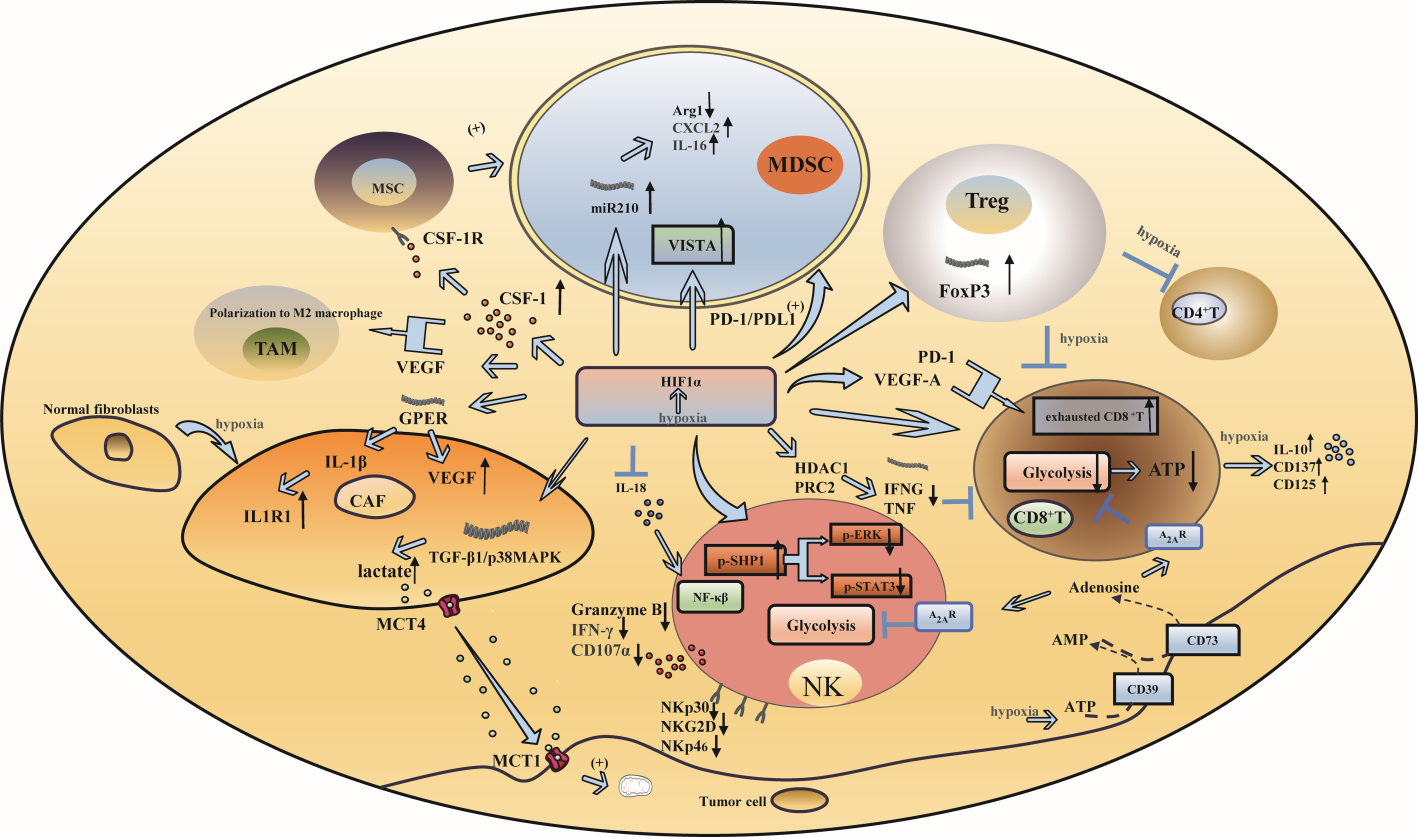
Hypoxia and immune escape in triple-negative breast cancer. **(A)** Hypoxia induces increased infiltration of immunosuppressive cells: Under hypoxic conditions, HIF-1α influences the function of MDSCs through microRNA-210, PD-L1 and VISTA. Additionally, HIF-1α enhances the generation of Tregs by upregulating FoxP3 transcription. **(B)** Hypoxia inhibits the activation and function of immune effector cells: The reduced expression of granzyme B, IFN-γ, and the degranulation marker CD107a, as well as the downregulation of activating receptors including NKP30, NKp46, and NKG2D on the surface of NK cells, resulted in a significant decrease in NK cell activity. The upregulation of HIF- α led to a reduction in glycolytic activity in CD8+ T cells. VEGF and PD-1, both regulated by HIF- α, influenced the anti-tumor response of CD8+ T cells. **(C)** Hypoxia can produce effects that affect mesenchymal cell function: CAF enhance the activity of cancer cell mitochondria by activating the TGF-β1/p38MAPK/MMP2/9 signaling axis to produce lactic acid under hypoxic conditions. **(D)** Hypoxia changes the relationship between cytokines and immune cells: HIF-1α has the ability to suppress the expression of interleukin-18 (IL-18), whereas hypoxia triggers the release of the immunosuppressive cytokine IL-10 and the upregulation of CD137 and CD25.

Hypoxia is a prominent characteristic of many solid tumors (21), particularly in TNBC (22, 23). Hypoxia causes an increase in hypoxia-inducible factor (HIF), which affects the tumor microenvironment, changes the metabolic state of tumors, and even promotes tumor angiogenesis by promoting the production and aggregation of immunosuppressive cells, inhibiting the function of immune effector cells, activating the signaling axis of stromal cells, and enhancing the relationship between cytokines and immune cells. This leads to the invasion and progression of TNBC (**Figure 1**).

### 2.1 Hypoxia induces increased infiltration of immunosuppressive cells

Myeloid-derived suppressor cells (MDSCs) inhibit immune responses and promote tumor immune escape (24). Under hypoxic conditions, hypoxia-inducible factor 1 α (HIF-1α) can synergize with chemokine signals from mesenchymal stem cells to trigger colony-stimulating factor 1 (CSF-1) and chemokine receptor type 5 (CCR5) gene transcription in breast cancer cells. Chemokine (C-C motif) ligand 5(CCL5) and CCR5 signaling regulates the expression of CSF1 in breast cancer cells, while CSF1 and CSF1 receptor (CSF1R) signaling regulates the expression of CCL5 in mesenchymal stem cells. This signaling cascade ultimately facilitates the recruitment of MDSCs (25).In addition, it was found that HIF-1α directly interacts with the HRE located in the proximal promoter region of programmed death-ligand 1 (PD-L1) and selectively upregulates PD-L1 on MDSCs. Inhibition of PD-L1 can abrogate MDSC-mediated suppression of T cell suppression in part by modulating the production of cytokines interleukin-6 (IL-6) and interleukin-10 (IL-10) in hypoxic MDSCs (26). Concurrently, HIF-1α upregulates the expression of microRNA 210 in MDSCs and then regulates the activity of MDSCs by regulating arginase-1 (ARG1), C-X-C motif chemokine ligand 12 (Cxcl12) and interleukin-16 (IL16), thus enhancing the immunosuppressive function of MDSCs (27). Furthermore, the V-domain Ig suppressor of T-cell activation (VISTA) is a mediator of MDSC function, and HIF-1α binds to the conserved hypoxia response element in the T-cell-activated VISTA promoter to upregulate VISTA in myelocytes. Targeting VISTA with antibodies or gene ablation can mitigate MDSC-mediated T-cell inhibition (28).

The expression of HIF-1α can promote the transcription of forkhead boxP3 (FoxP3), which in turn promotes the production of regulatory T cells (Tregs), thus strengthening their inhibitory function (29–31). Hypoxia can induce Tregs to inhibit the proliferation and differentiation of CD4^+^ T cells and CD8^+^ T cells (32, 33). Hypoxia induces a variety of cell types, including Tregs. In addition, hypoxia induces the expression of CD73 in various types of cells, including Tregs, which has a negative effect on T-cell function by participating in the production of adenosine, an immunosuppressive metabolite (34).

Hypoxia leads to the upregulation of HIF-1, which in turn promotes tumor angiogenesis and invasiveness, while tumor-associated macrophages (TAMs) play an important role in promoting tumor angiogenesis; therefore, HIF-1 is considered to be the factor by which TAMs promote angiogenesis (35, 36).

### 2.2 Hypoxia inhibits the activation and function of immune effector cells

Although natural killer (NK) cells play an important role in tumor immunity, decreased expression of granzyme B, interferon-gamma (IFN-γ), and the degranulation marker CD107a and reduced expression of activated receptors on the surface of NK cells, such as NKP30, NKp46 and NKG2D, can significantly reduce the activity of NK cells. Some studies have indicated that the decreased phosphorylation of extracellular signal-regulated kinase (ERK) and signal transducer and activator of transcription 3 (STAT3) induced by hypoxia is closely associated with the attenuation of NK cytotoxicity (37). Hypoxia can also promote the transformation of NK cells into tumor-resistant and immunosuppressive phenotypes. Because of the enrichment of the NF-kB pathway in NK cells, the antitumor activity of NK cells can be enhanced by inhibiting HIF-1α (38).

CD8^+^ T cells are critical antitumor immune cells. TNBC patients with high levels of CD8^+^ T cells exhibit improved clinical prognosis and a robust immune response (39). The immune-killing function of CD8^+^ T cells is inhibited in the hypoxic microenvironment, which is determined by the epigenetic mechanism of immune effector genes mediated by HIF-α (40). Tumor cells with elevated expression of HIF-α can consume large amounts of glucose and decrease the glycolytic activity of CD8^+^ T cells, resulting in a decrease in adenosine triphosphate (ATP), which in turn affects the function of CD8^+^ T cells (40, 41). The antitumor activity and infiltration level of CD8^+^ T cells are affected by vascular endothelial-derived growth factor (VEGF) and programmed cell death protein 1 (PD-1), which are regulated by HIF-α. VEGF-A can affect the transport and killing ability and promote the differentiation of PD-1^+^ TIM-3^+^ CXCR5^+^ terminally exhausted-like CD8^+^ T cells (42). An *in vitro* study of TNBC culture systems showed that HIF-1α inhibited the expression of immune effector genes such as interferon gamma (IFNG) and tumor necrosis factor (TNF) through histone modification mediated by histone deacetylase 1 (HDAC1) and polycomb repressive complex 2 (PRC2) during hypoxia and caused CD8^+^ T cells to dysfunction (43). Moreover, the amount of adenosine produced by tumor cells regulated by HIF-1α increased in the hypoxic microenvironment. Adenosine interacts with adenosine A2A receptors to promote T-cell apoptosis and inhibit proliferation, thereby suppressing antitumor immune function (44).

### 2.3 Hypoxia can affect mesenchymal cell function

Stromal cells include fibroblasts, astrocytes, adipocytes and other types, among which cancer-associated fibroblasts play an important role in hypoxia. Cancer-associated fibroblasts (CAFs) are highly sensitive to hypoxia, and fibroblast-specific HIF activates and promotes the arrangement and stiffness of the extracellular matrix (ECM), leading to morphological and migratory changes in breast cancer cells. During hypoxia, CAFs produce lactic acid, which is released by monocarboxylate transporter4 (MCT4) by activating the TGF-β1/p38MAPK/MMP2/9 signaling axis, and enters the cell with the help of the cancer cell monocarboxylate transporter 1 (MCT1). This process enhances the activity of cancer cell mitochondria and promotes cancer cell invasion (45). Hypoxia can also induce the transformation of normal fibroblasts into CAFs (46). In breast cancer, HIF-1α and the G protein-coupled estrogen receptor (GPER) signaling pathway stimulate the expression of VEGF in CAFs, while GPER in breast cancer CAFs induces interleukin-1β (IL-1β) to express interleukin-1 receptor 1 (IL1R1) in breast cancer cells (47). Therefore, by investigating the effect of GPER silencing on breast cancer caused by hypoxia, it was found that the expression of connective tissue growth factor (CTGF) could be inhibited by knocking down GPER in CAFs to inhibit the invasion of breast cancer cells induced by hypoxia (48).

### 2.4 Hypoxia changes the relationship between cytokines and immune cells

HIF-1α inhibits the production of interleukin-18 (IL-18), which is necessary for the activation of NF-κB and enhancement of the antitumor activity of NK cells (38). The oxygen tension during the activation of CD8^+^ T cells is decreased due to hypoxia, which in turn induces the secretion of the immunosuppressive factor IL-10 and the upregulation of CD137 and CD25. As a result, the phenotype of CD8^+^ T cells transitions from that of effector cells to that of nonproliferating cells (49). Simultaneously, hypoxia-induced production of VEGF, epithelial growth factor receptor (EGFR), C-C Motif Chemokine Ligand 5 (CCL5), CSF-1, oncostatin M (OSM), eotaxin, succinic acid and granulocyte-macrophage colony-stimulating factor (GM-CSF) can facilitate the recruitment of TAMs and promote their polarization toward the M2 phenotype (50–53).

## 3 Altered glycolysis and TNBC

**Figure 2:**
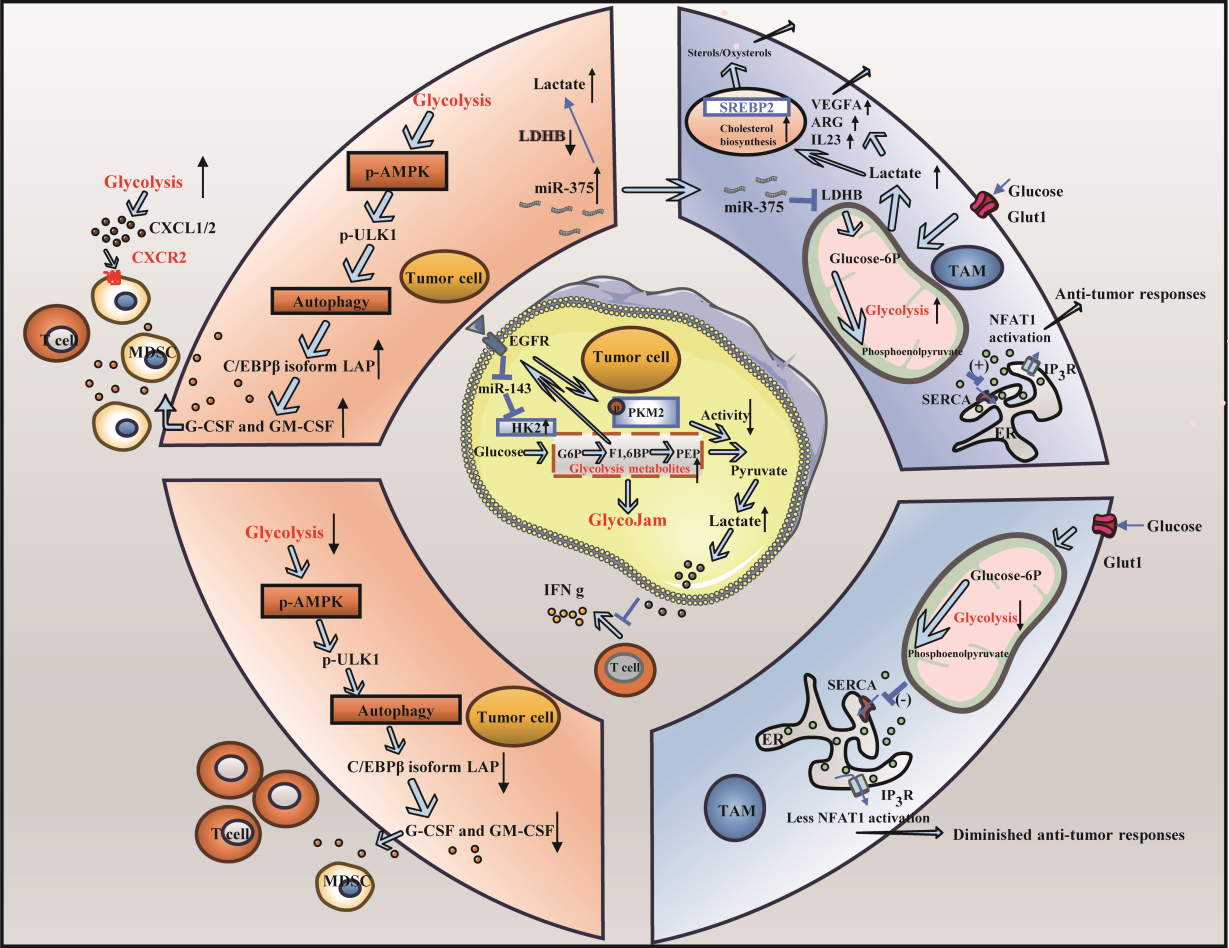
Altered glycolysis and triple-negative breast cancer. **(A)** The change of glycolysis rate is involved in immune escape of triple negative breast cancer: High rates of tumor cell glycolysis activate LAP leading to the formation of MDSCs and a decrease in the population of effector CD8+ T cells. Furthermore, T cells exhibited decreased glycolytic activity in the glucose-deficient tumor microenvironment, leading to impaired anti-tumor response. **(B)** Carbohydrate metabolite lactic acid participates in immune escape of triple negative breast cancer cells: Lactic acid produced by cancer cells induces the upregulation of VEGF and ARG1, thereby facilitating the differentiation of TAMs toward the M2 phenotype. Furthermore, the elevated lactic acid levels within TAMs result in reduced fatty acid levels, heightened cholesterol synthesis, and enhanced cancer cell proliferation.

In TNBC, tumor proliferation is closely related to glycolysis. When glycolysis is altered, the altered rate of glycolysis and the metabolites of glycolysis lead to changes in the nutrient supply to immune cells as well as to other cells, which can lead to problems such as the immune escape of tumor cells (54, 55) (**Figure 2**).

### 3.1 Changes in the glycolysis rate are involved in immune escape in TNBC

The modulation of the glycolysis rate can facilitate tumor immune escape. High rates of tumor cell glycolysis can activate the autophagy signaling pathway and AMP-activated protein kinase ULK1 (AMPK-ULK1), resulting in inhibition of enhancer-binding protein beta (CEBPB) subtypes and activation of liver-enriched activator protein (LAP), thus controlling the expression of GM-CSF and granulocyte colony-stimulating factor (G-CSF), supporting the formation of MDSCs, reducing the number of effector CD8^+^ T cells, weakening antitumor immunity, and hastening the progression of TNBC. Conversely, when glycolysis is limited, the expression of G-CSF and GM-CSF is inhibited, which affects the formation of MDSCs and increases the number of CD8^+^ T cells (56). In addition, during high rates of tumor cell glycolysis, high glucose and high oxygen consumption cause a glucose-deficient tumor microenvironment, which decreases the glycolytic ability of T cells, reduces the production of the metabolite phosphoenolpyruvate (PEP), and weakens the ability to inhibit the activity of sarco/ERCa^2 +^-ATPase (SERCA), resulting in a decrease in the ability of SERCA to maintain T-cell receptor-mediated Ca-NFAT signal transduction and effector function, thereby attenuating the antitumor response of T cells. Conversely, if tumor cells do not consume glucose excessively, the glycolytic capacity of T cells remains intact, and PEP, a glycolytic metabolite, ensures the antitumor function of T cells by inhibiting SERCA activity (57). Some studies have demonstrated that by inducing the Warburg effect in the tumor microenvironment (TME), activated C-X-C motif chemokine ligand 1/2 (CXCL1/2) binds to C-X-C motif chemokine receptor 2 (CXCR2), recruiting MDSCs and other cells, thereby fostering tumor growth and immune escape (58). Notably, during tumor glycolysis, lactate dehydrogenase indirectly modulates the activity of CD4^+^ T cells via the PD-1 pathway, depriving these cells of sufficient energy and consequently inhibiting their function (59).

### 3.2 Carbohydrate metabolite lactic acid participates in the immune escape of TNBC cells

The increase in glycolysis in TNBC cells results in the production of a large amount of lactic acid, which accumulates in the TME and forms an acidic environment (60). The function of immune cells, especially immunosuppressive TAMs, is inhibited by high levels of lactic acid and an acid-stimulated TME in tumors, which blocks the immune surveillance of tumors, resulting in immune escape. TAMs can differentiate and proliferate into a pretumor phenotype in this acidic environment, facilitating tumor immune escape (61). First, lactic acid from cancer cells can stimulate the expression of VEGF and ARG1 and promote the polarization of TAMs to the M2 phenotype through histone lactoacylation, leading to tumor cell proliferation (62–64). In addition, a large amount of lactic acid in TAMs is induced by downregulating lactate dehydrogenase B (LDHB), resulting in a decrease in fatty acid synthesis, which activates sterol regulatory element binding transcription factor 2 (SREBP2), increases cholesterol synthesis in TAMs, and increases cholesterol levels that promote cancer cell proliferation (44). Lactic acid can also activate the lactate receptor GPR81, which is related to tumor growth in dendritic cells, thereby eliminating antigen presentation, reducing the secretion of the proinflammatory cytokines IL-6 and IL-12, and inhibiting T-cell function (65). Some studies have shown that in TNBC, pyruvate kinase isozyme type M2 (PKM2) is suppressed through the EGFR signal transduction axis, and glucose phosphorylation is catalyzed by upregulating hexokinase 2 (HK2) to form a “glycolytic jam”, thus reducing the expression of INF-γ and IL-2, affecting T-cell function and contributing to tumor growth and immune escape (66).

Notably, as an intermediate of glycolysis, glucose-6-phosphate affects the antitumor immune response of immune cells through the pentose phosphate pathway (PPP) (67). This process interferes with oxidative phosphorylation (OXPHOS) and the PPP of immune cells, ultimately suppressing immune function (68, 69).

## 4 Lipid metabolism and TNBC

**Figure 3:**
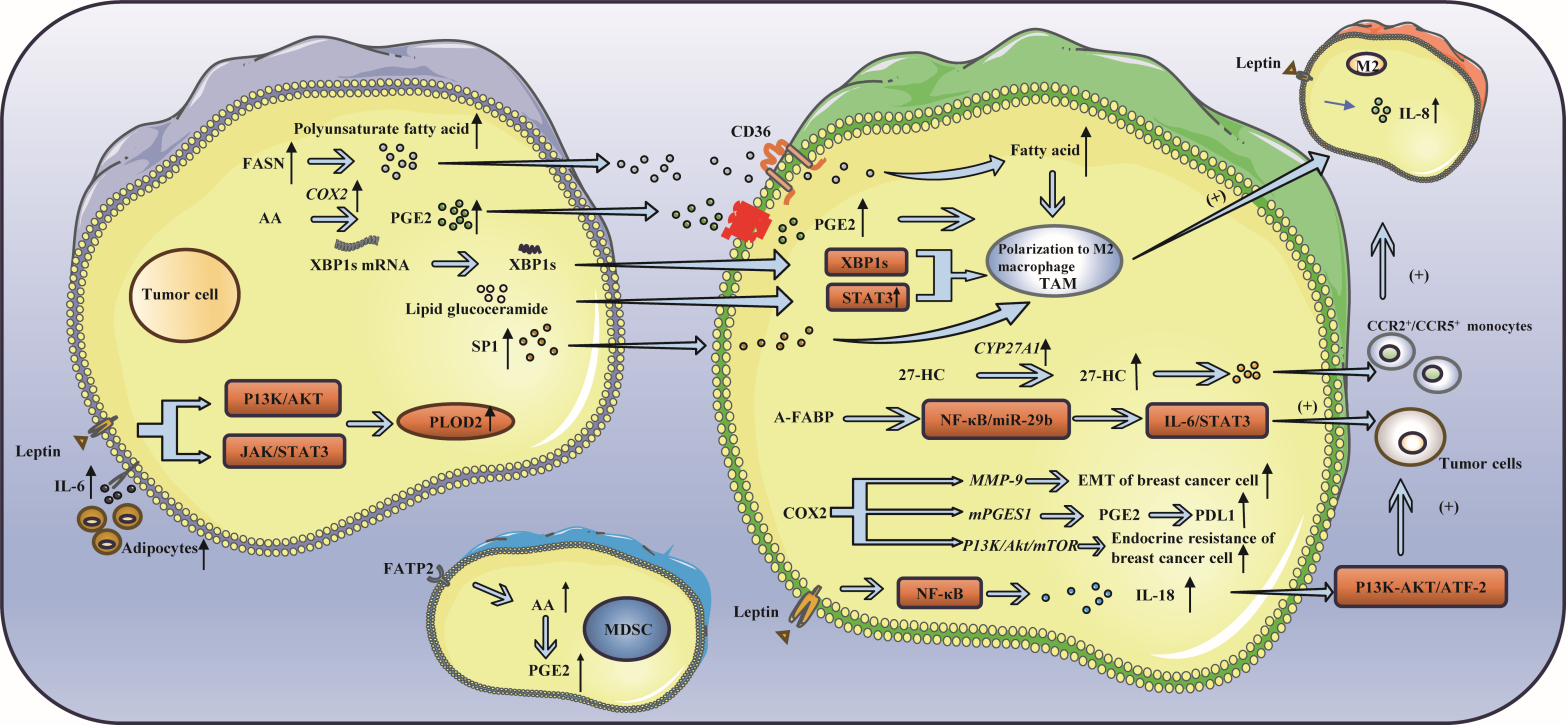
Lipid metabolism and triple-negative breast cancer. **(A)** Lipid metabolism can influence immune cell function: FASN and PGE2 in cancer cells have been shown to induce the polarization of TAMs toward the M2 phenotype. TAMs secrete 27-HC, which can enhance cancer cell proliferation and drive the differentiation of monocytes into M2 macrophages. Activation of IRE1 splicing pathway leads to the activation of signal transducer and activator of STAT3 and XBP1, thereby promoting the acquisition of a precancerous phenotype by TAMs. Additionally, S1P released from apoptotic tumor cells has been found to induce TAM-like polarization in macrophages. **(B)** Leptin, a fat factor in lipid metabolism, is involved in immune escape: Leptin has been shown to upregulate the expression of IL-8 in M2 macrophages and IL-18 in TAMs. Additionally, leptin has been found to increase the expression of PLOD2, thereby promoting cancer cell metastasis. Furthermore, leptin has the ability to suppress the function of CD8+ T cells, leading to immune escape.

During the occurrence and development of breast cancer, fatty acids can be absorbed by tumor cells, which in turn affects the absorption of fatty acids by immune cells. The activation, infiltration and effector function of immune cells are also disrupted by disordered lipid metabolism (70, 71), which in turn leads to immune escape (72–74) (**Figure 3**).

### 4.1 Lipid metabolism can influence immune cell function

The metabolism of lipids impacts immunosuppressive cells, such as M2 macrophages, through various pathways (75). TAM are commonly classified into two main phenotypes: the anti-tumor M1 and the pro-tumor M2. M2 macrophages rely on fatty acids as a source of energy to produce ATP, thereby promoting fatty acid oxidation (FAO) (76).The TME contains many signaling molecules that can alter lipid metabolism in TAMs by increasing FAO and lipid uptake, promoting TAM polarization to a tumor-promoting M2 phenotype, thereby exerting an immunosuppressive effect and promoting tumor growth, metastasis, and angiogenesis (77). In cancer cells, fatty acid synthase (FASN) increases polyunsaturated fatty acids and promotes the polarization of TAMs to the M2 phenotype under the regulation of CD36 (76, 78). Prostaglandin E2 (PGE2), which is derived from arachidonic acid in cancer cells, can also promote the polarization of TAMs to the M2 phenotype (79–81). 27-HC, a metabolite of cholesterol (CHOL) secreted by TAMs, can not only promote the proliferation of cancer cells but also stimulate TAMs to secrete chemokines, causing CCR2^+^ and CCR5^+^ monocytes to migrate to the tumor site and polarize into M2 macrophages (82). Moreover, the endoplasmic reticulum of macrophages can be induced by lipids in cancer cells to induce stress and activate STAT3 and X-box binding protein 1 (XBP1) through inositol-requiring enzyme 1 (IRE1) splicing, thus promoting the pretumor phenotype of TAMs (83). Moreover, apoptosis-derived sphingosine-1-phosphate (S1P) promotes TAM-like polarization of macrophages (84). The tumor-promoting factor adipose fatty acid-binding protein (A-FABP) is highly expressed in TAMs. A-FABP enhances IL6/STAT3 signal transduction, promoting cancer growth and metastasis by regulating the NF-κB/miR-29b pathway. The PGE2-COX2 (cyclooxygenase-2) axis plays an important role in the lipid metabolism of TAMs (85, 86). COX-2 in TAMs promotes epithelial–mesenchymal transition of BCCs by triggering the expression of matrix metalloproteinase 9 (MMP-9) (87). TAMs increase the expression of COX-2 through the PI3K/Akt/mTOR pathway, thus enhancing endocrine drug resistance in breast cancer (88), and the expression of PDL1 in TAMs can be regulated through the COX2/mPGES1/PGE2 pathway (89). Moreover, fatty acid transport protein 2 (FATP2) promoted the accumulation of arachidonic acid, resulting in increased prostaglandin E2 synthesis in MDSCs, thus enhancing the immunosuppressive activity of MDSCs (90). Due to the metabolism of tumor cells, the tumor microenvironment lacks nutrients and accumulates immunosuppressive metabolites, which leads to obvious inhibition of the tumor microenvironment, resulting in obvious inhibition of immune effector cells such as NK cells, even in a resting state (91, 92). In this state, lipid metabolism is enhanced (93, 94), and peroxisome proliferator-activated receptors drive lipid proliferation in NK cells, leading to complete “paralysis of cellular metabolism and transport (95, 96). Therefore, preventing lipids from entering the mitochondria can reverse the metabolic paralysis of NK cells and restore cell activity (75). Additionally, senescent CD8^+^ T cells accumulate continuously by the binding of cytosolic phospholipase A2 alpha (cPLA2a) to MAPK/STAT signals, which induces TNBC cells to undergo immune escape (97). Moreover, the ability of CAFs to take up exogenous lipids can be enhanced by the upregulation of FATP1, thus promoting the metastasis of cancer cells (98, 99).

### 4.2 Leptin, a fat factor involved in lipid metabolism, is involved in immune escape

There are more adipocytes around the tumor in patients with breast cancer, particularly in obese patients. Adipocytes can produce numerous adipokines, such as leptin, thus promoting tumor progression (100). First, leptin mediates the bidirectional interaction between cancer cells and TAMs (101). Leptin increases the expression of IL-8 in M2 macrophages and leads to the migration and invasion of cancer cells. Leptin can also promote the expression and secretion of IL-18 in TAMs through NF-κB/NF-κB1, thereby fostering the expression and proliferation of cancer cells through the PI3K-AKT/ATF-2 pathway (102). In addition, leptin and adipocyte-derived IL-6 enhance the expression of lysyl hydroxylase (PLOD2) by activating the JAK/STAT3 and PI3K/AKT signaling pathways, ultimately facilitating cancer cell metastasis (103–106). Some studies have indicated that leptin can be secreted by tumor-stromal adipocytes (TSAs) stimulated by external conditions and bind to receptors on tumor cells, resulting in proliferation, invasion and other effects (107, 108). Moreover, leptin can also accumulate in breast adipocytes and adipose tissue, activate the STAT3-FAO axis, reduce glycolysis, inhibit the function of CD8^+^ T cells, and cause immune escape (100).

Targeting fatty acid receptor CD36 may be effective against multiple cancers (109, 110).Inhibition of fatty acid binding proteins (FABPs) has demonstrated potential anti-tumor effects. In the prostate cancer, FABP5 has emerged as a novel therapeutic target (111).Of course, inhibition of FATP2 can also be considered (112).FASN inhibitors show significant anti-tumor effects in multiple cancers (113).Upstream regulators SREBPs are key to lipid synthesis and can be considered as potential therapeutic targets (114).Due to the plasticity of lipid metabolism, cancer cells can switch to another metabolic pathway when one pathway is blocked, which to some extent hinders the anti-tumor efficacy of monotherapy (115).Therefore, we can consider combining treatments to enhance the therapeutic effect.

## 5 Amino acid metabolism is involved in immune escape in TNBC

**Figure 4:**
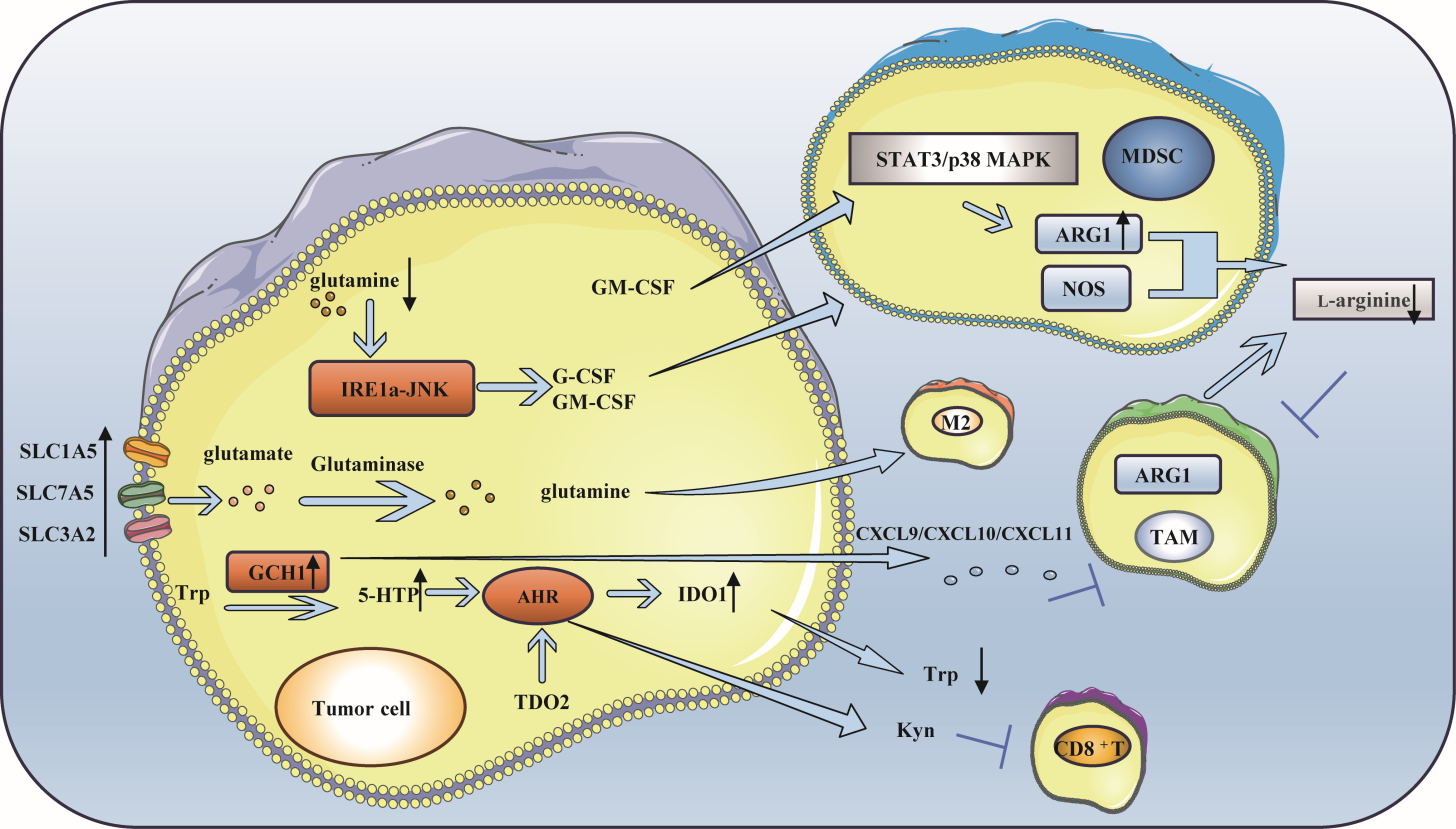
Amino acid metabolism is involved in immune escape of triple-negative breast cancer. **(A)** Glutamine metabolism participates in immune escape: A deficiency in glutamine leads to the production of MDSC, which in turn facilitates immune escape. Simultaneously, the breakdown of glutamine to produce a-KG is advantageous for sustaining the M2 phenotype of TAM. **(B)** Arginine metabolism participates in immune escape: L-arginine can hydrolyze and lead to the lack of available arginine in the microenvironment, which inhibits the function of immune cells. ARG 1 and NOS activated in tumor microenvironment inhibit NO production and promote tumor development. L-Arg is consumed by MDSCs, which inhibits T cell activation. Increased expression of ARG1 in breast cancer causes TAM to differentiate into M2 phenotype. **(C)** Tryptophan metabolism is involved in immune escape: GCH1 is highly expressed in TNBC, resulting in a low tryptophan microenvironment and immune escape. In addition, the activity of TDO2 in TNBC cells increased, which produced kynurenine and inhibited the viability of CD8+T cells.

Amino acids are essential nutrients for cells, and the demand for amino acids in normal cells is lower than that in tumor cells (116). Like glucose, amino acids are important fuels for the development of tumor cells. For example, glutamine metabolism in breast cancer can maintain the balance between Tregs and effector T cells (Teffs), change the function and state of immune cells in the TME, control the level of reactive oxygen species (ROS), provide the energy needed for immune cell progression, and lead to immune escape (117). Arginine is the basis of protein synthesis and the premise of metabolism and is mainly involved in the activation and functional activation of immune cells, especially T cells. In addition, tryptophan is an essential amino acid for cell proliferation, and its catabolism can inhibit T-cell proliferation (118). Thus, they are particularly important in the metabolism of TNBC (**Figure 4**).

### 5.1 Glutamine metabolism participates in immune escape

Although glutamine is a nonessential amino acid (NEAA), many tumor cells show a “glutamine dependence” in the TME (119, 120). The enhancement of glutamine metabolism in breast cancer alters the function and state of immune cells within the TME, which plays a crucial role in maintaining the balance between Teffs and Tregs and in immune escape (121–125). Research has shown that when glutamine deficiency occurs, G-CSF and GM-CSF are expressed in breast cancer cells through the IRE1 α-JNK pathway to induce the production of MDSCs, resulting in immune escape (126). Moreover, the overexpression of glutamine transporter proteins (such as recombinant solute carrier family 1, member 5 (SLC1A5), SLC7A5, and SLC3A2) in cancer cells directly influences glutamine metabolism, produces specific subtypes of inflammatory infiltration, and impacts the differentiation of TAMs toward a pro-carcinogenic phenotype (121). The α-ketoglutarate (α-KG) generated during glutamine catabolism supports the maintenance of a TAM M2-like phenotype (127, 128).

### 5.2 Arginine metabolism participates in immune escape

One of the key enzymes involved in arginine metabolism is ARG1, which is highly expressed in TNBC (129). Arginase plays a crucial role in promoting immune escape through several mechanisms: The content of L-arginine is closely related to the survival and proliferation of T cells (130–132). L-arginine is hydrolyzed to L-ornithine and urea under the catalysis of ARG1, which leads to a lack of available arginine in the microenvironment (104), which suppresses the function of immune cells and results in immune escape. Activated ARG1 and nitric oxide synthase (NOS) in the tumor microenvironment affect TAMs and inhibit the production of nitric oxide (NO), which can promote the differentiation of M1 macrophages, thus facilitating tumor development. The coexpression of ARG1 and inducible nitric oxide synthase (iNOS) enhances the production of ROS and reactive nitrogen species, which further inhibits the function of T cells within tumor cells (133, 134). The kinase GCN affects MDSCs during l-arginine depletion, and the L-arginine (L-Arg) is consumed by NOS and ARG-1 produced by MDSCs through the kinase GCN2, leading to amino acid starvation, thus inhibiting T-cell activation. MDSCs disrupt innate immunity and affect antitumor immunity by interacting with macrophages, NK cells, and NK T cells (135). In breast cancer, the expression of ARG1 increases when GM-CSF stimulates the STAT3 or p38MAPK signaling pathway, and immune cells in the microenvironment obtain less L-arginine, which increases the phenotypic differentiation of TAMs to M2 macrophages, reduces the infiltration of CD8^+^ T cells and ultimately promotes immune escape (134).

### 5.3 Tryptophan metabolism is involved in immune escape

Tryptophan catabolism plays an important role in immune escape in TNBC (118, 136). Its metabolism is closely associated with enzymes such as indoleamine 2,3-dioxygenase (IDO) and tryptophan 2,3-dioxygenase (TDO) (137–141). Guanosine triphosphate cyclohydrolase 1 (GCH1), which is highly expressed in TNBC, regulates the metabolism of tryptophan (Trp), increases the level of 5-hydroxytryptophan (5-HTP), activates the aryl hydrocarbon receptor (AHR), and enhances IDO1 activity. This leads to a reduction in tryptophan levels and an increase in kynurenine levels. It is worth noting that kynurenine plays a role in promoting immunosuppression in the immune system. In addition, IDO1 can upregulate FoxO3α by activating Tregs, subsequently upregulating PD-1 and inducing sustained immune suppression via the PD-1/PTEN pathway, ultimately resulting in immune escape (142–145). Moreover, some studies have suggested that GCH1 mainly induces immunosuppression in an IDO1-dependent manner; however, the expression of chemokines such as CXCL9, CXCL10 and CXCL11 decreases because of the expression of GCH1, an increase in the tryptophan metabolite kynurenine and a decrease in tryptophan, which leads to a decrease in effector T-cell aggregation and promotes immune escape (146). Other studies have shown that IDO in breast cancer mediates MDSC-induced T cell proliferation and Th1 polarization inhibition, promotes T cell apoptosis and the secretion of immunosuppressive cytokines (IL-10 and TGF-β), causing breast cancer immune escape (147). Notably, when IDO is overexpressed, TAMs tend to polarize to the M2 phenotype, and T-cell activity is inhibited (148). In addition, the increased activity of TDO2 in TNBC cells mediates the initial production of the tryptophan catabolite kynurenine by AhR, which hinders the viability of CD8^+^ T cells and facilitates tumor immune escape (149, 150). Ultimately, by depleting tryptophan, the tryptophan metabolite kynurenine accumulates, inhibiting the function of effector T cells and NK cells and stimulating Tregs, MDSCs and macrophages to polarize into tolerant phenotypes, resulting in an immunosuppressive TME (151).Overall, IDO1 promotes immunosuppression through tryptophan depletion and direct effects of tryptophan catabolites. However, the interaction between TDO and other immune cells remains unclear, and the specific role of TDO in regulating immune responses is not well understood (152, 153).

## 6 Microbiome and TNBC

The microbiome can influence tumor development and progression (154). Current research has primarily concentrated on bacterial communities within tumors. Study finds potential link between tumor microbiome and cancer development (155, 156). The intratumoral microbiota is currently shown to play an important role in the pathogenesis of cancer (157). Studies have found that the toxic protein BFT-1 secreted by enterotoxigenic Bacteroides fragilis can promote the lysosomal degradation of NUMB by binding to NOD1, which is highly expressed on breast cancer stem cells, thereby activating the NOTCH1-HEY1 pathway, promoting breast cancer cell stemness and chemotherapy resistance to docetaxel (158). In contrast, trimethylamine N-oxide (TMAO), a metabolite related to the genera under Clostridiales, was more abundant in tumors with an activated immune microenvironment. Trimethylamine N-oxide induces tumor cell pyroptosis by activating endoplasmic reticulum stress kinase PERK, thereby enhancing CD8^+^ T cell-mediated anti-tumor immunity in TNBC. The results of this study suggest that microbial metabolites may serve as a new therapeutic approach to improve the efficacy of immunotherapy for TNBC (159). Tumor-associated microbiota plays an important role in the occurrence and development of tumors. Researchers have currently conducted relevant research on the carcinogenic mechanism of the microbiome, but more research is still needed to further explain it. It is worth noting that the intestinal microbiota is a research hotspot, which leads to insufficient understanding of related contents such as the microbiota of other ecological niches, so there is still a long way to go for future research (160).

## 7 Concluding remarks

In summary, metabolic reprogramming plays a pivotal role in the development and progression of TNBC and is one of the hallmarks of tumor progression. Under hypoxia, the expression of granzyme B, IFN-γ and degranulation marker CD107a on the surface of NK cells decreased, and the expression of activated receptors NKP30, NKP46 and NKG2D on NK cells decreased, resulting in a significant decrease in the activity of NK cells. In addition, HIF-1 α is one of the important factors. HIF-1 α can affect the function of MDSCs through microRNA210, PD-L1 and VISTA, up-regulate the transcription of FoxP3 to enhance the production of Treg, and inhibit the expression of IL-18. High rates of tumor cell glycolysis activate LAP leading to the formation of MDSCs and a decrease in the population of effector CD8+ T cells. At the same time, lactic acid produced by cancer cells up-regulated VEGF and ARG1, promoting TAM differentiation into the M2 phenotype. With the change of lipid metabolism, FASN and PGE2 in cancer cells can induce TAM to M2 phenotypic polarization, while 27-HC secreted by TAMs can promote cancer cell proliferation and promote monocytes to differentiate into M2 macrophages. In addition, Leptin has been shown to upregulate the expression of IL-8 in M2 macrophages and IL-18 in TAMs, increase the expression of PLOD2, and promote the metastasis of cancer cells. Arginine plays an indispensable role in amino acid metabolism and the lack of available arginine in the microenvironment inhibits immune cell function. L-arginine, absorbed by bone marrow mesenchymal stem cells, inhibits T cell activation. The high expression of ARG1 in breast cancer leads to TAM differentiation into the M2 phenotype. Tryptophan is also involved in immune escape, with GCH1 highly expressed in TNBC resulting in a low tryptophan microenvironment and immune escape.

It is evident that metabolic reprogramming plays a crucial role in immune escape of TNBC. Due to the characteristics of high invasiveness, easy metastasis, and recurrence of TNBC, studying metabolic reprogramming is of great significance and can provide new insights for TNBC immunotherapy. With the widespread use of immunotherapy, drug resistance will be inevitable. Therefore, conducting thorough research on the tumor microenvironment and immune escape mechanisms, as well as elucidating the impact of metabolic reorganization on immune escape in TNBC, will serve as a crucial foundation for addressing drug resistance. We can identify metabolic-related targets that contribute to immune escape more precisely. By utilizing gene editing techniques, it may be possible to modify or decrease the metabolic factors responsible for immune escape, ultimately enhancing the effectiveness of immunotherapy in treating TNBC. During the patient’s treatment, finding metabolic level-related changes or targets can increase the synergistic effect through combined immunotherapy. More research is needed in the future to explore the potential of combined therapy and how to translate these mechanisms into effective clinical treatments. Through continued efforts and collaboration, more effective treatment options are expected to be provided to improve the prognosis of patients with triple-negative breast cancer.

## Glossary

**Table:**

TNBC	Triple-negative breast cancer
HIF	hypoxia-inducible factor
HIF-1α	hypoxia-inducible factor 1 α
MDSCs	myeloid-derived suppressor cells
CSF-1	colony-stimulating factor 1
PD-L1	programmed cell death1 ligand 1
ARG1	regulating arginase-1
Cxcl12	C-X-C motif chemokine ligand 12
IL16	interleukin-16
VISTA	V-domain Ig suppressor of T-cell activation
FoxP3	forkhead boxP3
Tregs	regulatory T cells
TAM	tumor-associated macrophages
NK	natural killer
IFN-γ	interferon-gamma
ERK	extracellular signal-regulated kinase
STAT3	signal transducer and activator of transcription 3
ATP	adenosine triphosphate
VEGF	vascular endothelial-derived growth factor
PD-1	programmed cell death protein 1
IFNG	interferon gamma
TNF	tumor necrosis factor
HDAC1	histone deacetylase 1
PRC2	polycomb repressive complex 2
CAF	Cancer-associated fibroblasts
ECM	extracellular matrix
MCT4	monocarboxylate transporter4
MCT1	monocarboxylate transporter 1
GPER	G protein-coupled estrogen receptor
IL-1β	interleukin-1β
IL1R1	interleukin-1 receptor 1
CTGF	connective tissue growth factor
IL-18	interleukin-18
EGFR	epithelial growth factor receptor
CCL5	C-C Motif Chemokine Ligand 5
OSM	oncostatin M
GM-CSF	granulocyte-macrophage colony-stimulating factor
AMPK-ULK1	AMP-activated protein kinase ULK1
CEBPB	enhancer-binding protein beta
LAP	liver-enriched activator protein
G-CSF	granulocyte colony-stimulating factor
PEP	phosphoenolpyruvate
SERCA	sarco/ERCa^2 +^-ATPase
TME	tumor microenvironment
CXCL1/2	C-X-C motif chemokine ligand 1/2
CXCR2	C-X-C motif chemokine receptor 2
LDHB	lactate dehydrogenase B
SREBP2	sterol regulatory element binding transcription factor 2
PKM2	pyruvate kinase isozyme type M2
HK2	hexokinase 2
PPP	pentose phosphate pathway
OXPHOS	oxidative phosphorylation
VLDL	very low-density lipoprotein
LDL	low-density lipoprotein
FAO	fatty acid oxidation
FASN	fatty acid synthase
PGE2	Prostaglandin E2
CHOL	cholesterol
XBP1	X-box binding protein 1
IRE1	inositol-requiring enzyme 1
S1P	sphingosine-1-phosphate
A-FABP	fatty acid-binding protein
MMP-9	matrix metalloproteinase 9
FATP2	fatty acid transport protein 2
cPLA2a	cytosolic phospholipase A2 alpha
PLOD2	lysyl hydroxylase
TSA	tumor-stromal adipocytes
Teffs	effector T cells
ROS	reactive oxygen species
NEAA	nonessential amino acid
SLC1A5	recombinant solute carrier family 1, member 5
α-KG	α-ketoglutarate
NOS	nitric oxide synthase
NO	nitric oxide
L-Arg	L-arginine
IDO	indoleamine 2, 3-dioxygenase
TDO	tryptophan 2, 3-dioxygenase
GCH1	guanosine triphosphate cyclohydrolase 1
Trp	tryptophan
5-HTTP	5-hydroxytryptophan
AHR	aryl hydrocarbon receptor.
